# Supplementary material for: Behavioral Changes, Adaptation, and Supports among Indonesian Female Sex Workers Facing Dual Risk of COVID-19 and HIV in a Pandemic
Source: Int J Environ Res Public Health. 2022 Jan 26;19(3):1361. doi: 10.3390/ijerph19031361 (PMC8835319; doi:10.3390/ijerph19031361)
Supplement: Supplementary file 1 [file ijerph-19-01361-s001.zip › ijerph-1506050-supplementary.pdf]

Table S1. Distribution and factor loading of sex work-related COVID-19 preventive behavior score.

Items	Distribution, n (%)				Loading Factor		Internal Reliability	
	Never	Sometime	Often	Always	Component I	Component II	Cronbach α	If item deleted
Prepare sanitation equipment before sex	46 (4.8)	120 (12.9)	324 (34.1)	461 (48.5)	**0.786**	0.193	0.915	0.905
Hand washing before sex	50 (5.3)	134 (14.1)	359 (37.7)	408 (42.9)	**0.804**	0.208		0.901
Check client's temperature	87 (9.1)	144 (15.1)	311 (32.7)	409 (43.0)	**0.822**	0.199		0.896
Check client for any symptoms	109 (11.5)	135 (14.2)	302 (31.8)	405 (42.6)	**0.811**	0.175		0.901
Mandatory shower before sex	52 (5.5)	120 (12.6)	315 (33.1)	464 (48.8)	**0.857**	0.151		0.895
Mandatory shower after sex	41 (4.3)	95 (10.0)	323 (34.0)	492 (51.7)	**0.835**	0.150		0.899
Wear face mask during sex	206 (21.7)	200 (21.0)	214 (22.5)	331 (34.8)	0.385	**0.720**	0.842	0.865
Wear face-shield during sex	383 (40.3)	267 (28.1)	125 (13.1)	176 (18.5)	0.154	**0.910**		0.708
Wear gloves during sex	464 (48.8)	247 (26.0)	107 (11.3)	133 (14.0)	-	**0.903**		0.760
**Eigen value**					4.952	1.617		
**Variance explained (%)**					55.03	17.97		
**Cumulative (%)**					55.03	73.00		

Table S2. Multivariate analysis for types of online sex work as determinants of major income reduction and reduced condom use.

Variables (n = 951)	Major Income Reduction		Reduced Condom Use	
	aOR (95% CI)	P	aOR (95% CI)	P
**Location, n (%)**				
**Bandung (n = 250)**	Ref.		Ref.	
**Greater Jakarta (n = 334)**	1.08 (0.73 – 1.61)	0.004**	0.64 (0.37 – 1.11)	0.054
**Yogyakarta (n = 152)**	2.81 (1.56 – 5.06)		0.32 (0.14 – 0.75)	
**Bali (n = 215)**	1.29 (0.83 – 2.02)		0.58 (0.30 – 1.11)	
**Age (years)**	1.02 (0.99 – 1.05)	0.176	1.00 (0.96 – 1.04)	0.988
**Education**				
**Not completed high school (n = 430)**	-	-	-	-
**High school (n = 480)**				
**College degree (n = 41)**				
**Employment other than sex work**				
**None (n = 606)**	Ref.	0.006**	-	-
**Employed (n = 345)**	0.62 (0.45 – 0.88)			
**Marital status, n (%)**				
**Single (n = 388)**	Ref.		Ref.	
**Cohabitation (n = 49)**	1.78 (0.84 – 3.79)	0.392	0.83 (0.32 – 2.15)	0.056
**Married (n = 148)**	0.86 (0.53 – 1.42)		0.76 (0.40 – 1.46)	
**Widowed/divorced (n = 366)**	1.00 (0.67 – 1.47)		0.43 (0.23 – 0.79)	
**Housing**				
**Alone (n = 355)**	-	-	-	-
**With roommate (n = 200)**				
**With family (n = 396)**				
**HIV status**				
**Negative (n = 488)**	Ref.	< 0.001**	Ref.	0.035*
**Unknown (n = 376)**	0.58 (0.42 – 0.82)		1.62 (1.00 – 2.62)	
**Positive (n = 87)**	0.20 (0.12 – 0.34)		2.44 (1.11 – 5.38)	
**Fear of COVID-19 score**				
**Low (n = 493)**	Ref.	< 0.001**	-	-
**High (n = 458)**	1.87 (1.36 – 2.57)			
**Engaged in video Call Sex**				
**No (n = 707)**	Ref.	0.042*	Ref.	0.803
**Yes (n = 244)**	0.69 (0.48 – 0.99)		1.06 (0.65 – 1.74)	
**Engaged in phone Call Sex**				
**No (n = 841)**	-	-	Ref.	0.038*
**Yes (n = 110)**			1.89 (1.04 – 3.46)	
**Engaged in chat Sex/Sexting**				
**No (n = 882)**	Ref.	0.663	-	-
**Yes (n = 69)**	0.88 (0.49 – 1.58)			
**Sanitation & personal hygiene during sex work, n (%)**				
**Low adherence (n = 477)**	Ref.	0.047*	Ref.	0.001**
**High adherence (n = 474)**	1.39 (1.00 – 1.92)		0.40 (0.24 – 0.65)	
**PPE use during sex work, n (%)**				
**Low adherence (n = 366)**	-	-	Ref.	0.499
**High adherence (n = 585)**			1.19 (0.72 – 1.96)	
**Client frequency changes**				
**Little to no effect (n = 306)**	Ref.	< 0.001**	-	-
**Major reduction (n = 645)**	3.30 (2.40 – 4.54)			
**Changes to condom access**				
**No change or easier (n = 792)**	N/A	N/A	Ref.	< 0.001**
**More difficult (n = 159)**			10.76 (6.71 – 17.76)	
